# Allelic Variation in Protein Tyrosine Phosphatase Receptor Type-C in Cattle Influences Erythrocyte, Leukocyte and Humoral Responses to Infestation With the Cattle Tick *Rhipicephalus australis*

**DOI:** 10.3389/fimmu.2021.675979

**Published:** 2021-07-09

**Authors:** Nicholas N. Jonsson, David K. Cox, Emily K. Piper, Emily F. Mantilla Valdivieso, Constantin Constantinoiu, Louise A. Jackson, Michael J. Stear, Elizabeth M. Ross, Ala E. Tabor

**Affiliations:** ^1^ Institute of Biodiversity Animal Health and Comparative Medicine, University of Glasgow, Glasgow, United Kingdom; ^2^ School of Veterinary Science, The University of Queensland, Brisbane, QLD, Australia; ^3^ Centre for Animal Science, Queensland Alliance for Agriculture and Food Innovation, The University of Queensland, Brisbane, QLD, Australia; ^4^ College of Public Health, Biomedical and Veterinary Sciences, James Cook University, Townsville, QLD, Australia; ^5^ Biosecurity Sciences Laboratory, Biosecurity Queensland, Department of Agriculture and Fisheries, Queensland Government, Brisbane, QLD, Australia; ^6^ Department of Animal, Plant and Soil Sciences, La Trobe University, Melbourne, VIC, Australia

**Keywords:** ticks & TBDs, host resistance, immunity, parasite, immunoglobulin, erythron, leukocytes

## Abstract

The protein tyrosine phosphatase receptor type-C (*PTPRC*) gene encodes the common leukocyte antigen (CD45) receptor. CD45 affects cell adhesion, migration, cytokine signalling, cell development, and activation state. Four families of the gene have been identified in cattle: a taurine group (Family 1), two indicine groups (Families 2 and 4) and an African “taurindicine” group (Family 3). Host resistance in cattle to infestation with ticks is moderately heritable and primarily manifests as prevention of attachment and feeding by larvae. This study was conducted to describe the effects of *PTPRC* genotype on immune-response phenotypes in cattle that display a variable immune responsiveness to ticks. Thirty tick-naïve Santa-Gertrudis cattle (a stabilized composite of 5/8 taurine and 3/8 indicine) were artificially infested with ticks weekly for 13 weeks and ranked according to their tick counts. Blood samples were taken from control and tick-challenged cattle immediately before, then at 21 d after infestation and each subsequent week for 9 weeks. Assays included erythrocyte profiles, white blood cell counts, the percentage of cellular subsets comprising the peripheral blood mononuclear cell (PBMC) population, and the ability of PBMC to recognize and proliferate in response to stimulation with tick antigens *in vitro*. The cattle were *PTPRC* genotyped using a RFLP assay that differentiated Family 1 and 3 together (220 bp), from Family 2 (462 bp), and from Family 4 (486 bp). The *PTPRC* allele frequencies were Family 1/3 = 0.34; Family 2 = 0.47; Family 4 = 0.19. There was no significant association between *PTPRC* genotype and tick count. Each copy of the Family 1/3 allele significantly decreased total leucocyte count (WCC) and CD8^+^ cells. Increasing dosage of Family 2 alleles significantly increased red blood cell count (RCC), haematocrit (PCV), and haemoglobin (Hb) concentration in blood. Increasing dosage of the Family 4 allele was associated with increased WCC, reduced RCC, reduced PCV and reduced Hb. Homozygote Family 1/3 animals had consistently lower IgG1 in response to tick Ag than homozygote Family 2 animals. The *PTPRC* genotype influences the bovine immune response to ticks but was not associated with the observed variation in resistance to tick infestation in this study.

## Introduction

*PTPRC* or protein tyrosine phosphatase receptor-type C, also known as CD45, or leukocyte common antigen (LCA) is a key component of the signal transduction cascade in immune cells (1). Throughout this report, we refer to *PTPRC* as the gene encoding CD45, although the gene as annotated for human and mouse has several aliases: *B220*, *CD45*, *CD45R, Cd45, GP180, LCA*, *L*-*CA, Ly-, LY5*, *Ly-5, Lyt-, Lyt-4,T200*. CD45 was initially investigated in cattle for its potential involvement in pathogen tolerance in African cattle (2). They found that allelic polymorphisms in CD45 constituted the basis for differential antibody staining in peripheral blood leukocytes from cattle of African, European, and Indian origin, and suggested that polymorphism might be associated with tolerance to regionally endemic pathogens.

CD45 is an abundant cell surface glycoprotein found in the plasma of all nucleated hematopoietic cells and controls the immune response by dephosphorylating molecules that initiate antigen receptor signalling in T- and B-cell cells, such as the Src family kinases (SFKs) (3, 4). There are many isoforms of differing molecular weight due to the alternative splicing of exons 4, 5 and 6 (referred to as A, B and C) in the extracellular domain. The smallest isoform is CD45RO of approximately 180 kDa, lacking all of the alternatively spliced exons, whereas the largest isoform that includes all three exons – CD45RABC is about 240 kDa and heavily glycosylated (1, 3, 5). In addition to these variably spliced domains, the protein comprises three fibronectin type III (FN3) repeats, a short transmembrane domain, and a cytoplasmic region of two tandemly duplicated PTPase homology domains (D1 and D2), in which only D1 is catalytically active (3). The expression of *PTPRC* is tightly regulated depending on the cell type, maturation, and activation state. Although nucleotide sequence in the extracellular domains is highly variable, the isoform structures are largely conserved across species (3, 6). In *Bos taurus* cattle, *PTPRC* is on chromosome 16, has at least 30 exons and nine characterized isoforms (Gene ID: 407152, NCBI, 2021). Human and *B. taurus PTPRC* sequences show approximately 70% sequence identity. In humans five CD45 isoforms are well characterized (6). Ballingall et al. (2) initially considered *PTPRC* as one of several genes that might influence the diverse responses of African and Asian cattle to endemic pathogens in Africa. They noted that peripheral blood leukocytes from African and European taurine cattle had similar CD45RO antibody staining patterns whereas in indicine cattle, the pattern was variable. The pattern of staining corresponded with four distinct allelic families of *PTPRC*: *B. taurus*, *Bos indicus* (×2), and cattle of African origin (2, 7).

Ballingal et al. (2) showed that there appeared to be strong natural selection on extracellular domains of CD45 protein and proposed that it was likely to be a determinant of the immunity of cattle to endemic pathogens. Loss-of-function mutations of *PTPRC* have consequences related to immunodeficiency and malignancy in humans and mice (4) and CD45 has been associated with disease in cattle. A microarray-based study showed that *PTPRC* expression in the mesenteric lymph nodes of cattle with high resistance to gastrointestinal nematodes was increased, which was subsequently confirmed by qRT PCR (8). In a study on the reactivity of subsets of leukocytes present in the skin of *B. taurus* and *B. indicus* cattle infested with *R. australis*, antibodies specific for CD45 and CD45RO epitopes bound differentially in taurine and indicine cattle (9). In a follow-up study using tick resistant and susceptible Santa Gertrudis cattle, the reactivity of cells to CD45 and CD45RO mAbs also differed between resistant and susceptible cattle of the same breed (10). It was proposed that CD45 variants of *B. indicus* lack the epitopes recognized by mAb raised against CD45 and CD45RO in taurine cattle, and that CD45 might therefore have potential as a biomarker for resistance to infestation with cattle ticks.

We hypothesised that sequence variation in *PTPRC* in cattle affects resistance to ticks and immune phenotype. Our aim here was to take observations on erythrocytes, leukocytes and immunoglobulins obtained from cattle that were experimentally infested with *R. australis* in a previous experiment (10, 11), genotype the animals for the major *PTPRC* variants, and determine whether variation in these observations was associated with the presence of *PTPRC* variants.

## Materials and Methods

### Background Experimental Design, Animals, Tick-Counts, and Immunological Assays

The experimental methods are described in detail in the earlier articles (10, 11) and summarized briefly here. Thirty-five tick-naïve Santa-Gertrudis cattle (a stabilized composite of 5/8 taurine – Shorthorn – and 3/8 indicine – Brahman) were used in this study, conducted near Brisbane, in Queensland, Australia. The cattle were from a single property of origin and were selected such that their parentage was as far as possible an even admixture of sires. Five cattle were held as control animals on a separate, tick-secure property within 5 km of the experimental farm, and the remaining 30 were artificially infested by application to the neck and withers of 10 000 (0.5 g) *Rhipicephalus australis* tick larvae weekly for 13 weeks. Tick larvae were of the Non-Resistant Field Strain (NRFS) that is maintained free of *Babesia* and *Anaplasma* pathogens at the Queensland Department of Agriculture and Fisheries’ Biosecurity Science Laboratories (12). Tick counts were conducted weekly using the standard tick count method of Utech et al. (13, 14). Each infestation consisted of larvae applied to the neck and withers. Blood samples were taken from control and tick-challenged cattle immediately before the first infestation, then at 21 d post primary infestation (PPI) and each subsequent week for 9 weeks. The study was conducted with approval from the University of Queensland Animal Ethics - Production and Companion Animals Committee (Approval numbers: SVS/864/06/CRC and SVS/872/07/CRC).

Tick count data recorded over 13 weeks were originally analysed using a mixed effects model applied to data summarized over time (median, area under the curve, final count) fit by restricted maximum likelihood (REML), to rank each animal on its ability to resist tick infestation.

Erythrocyte profiles and white blood cell counts were conducted using a VetABC animal blood cell counter (ABX Hematologie). The percentages of cellular subsets comprising the peripheral blood mononuclear cell (PBMC) population were determined using the Ab listed in **Table 1** with a FACSCalibur flow cytometer (Becton Dickinson Immunocytometry Systems), as described in detail by Piper et al. (15). The ability of PBMC to recognize tick antigen (Ag) and proliferate in response to stimulation with antigens *in vitro* was quantified for concanavalin-A (ConA), and Ag mixtures derived from soluble fractions of salivary gland (SS), mid-gut (GS) or larvae (LS), or membrane-bound fractions of salivary gland (SM) or mid-gut (GM) in triplicate using the method described by Piper et al. (11). Results of PBMC proliferation are expressed in terms of optical density (OD) of microplate photometric readings at 450 nm. IgG1 and IgG2 responses to tick infestation were conducted in triplicate using an indirect ELISA, in wells coated with fractionated tick Ag (salivary soluble – SS; gut membrane – GM; gut soluble – GS; larval soluble – LS) as described in detail in Piper et al. (15). Microtiter plates were coated with diluted tick antigens. Sera were diluted and added to the microtiter plates in triplicate. Monoclonal antibodies (mouse anti-bovine IgG1 and mouse anti-bovine IgG2) were added to all wells. The conjugated antibody (goat anti-mouse IgG heavy and light chain specific, conjugated to horseradish peroxidase) was then added to each well. A tetramethylbenzidine-peroxidase substrate was used to develop the signal, and the reaction was stopped with orthophosphoric acid. The absorbance was read at 450 nm and the mean OD of each biological sample from triplicate wells was used for statistical analysis.

**Table 1:** Monoclonal antibodies used and the cell subsets labelled in flow cytometric analysis of cellular subsets.

Specificity	Cell Subset	Identity	Source	Isotype
Isotype control		IgG1	Dako	IgG1
CD3	T cells	IgG1	VMRD^b^	
CD4	T helper	IL-A11	Cell culture^a^	IgG2a
CD8	T cytotoxic	IL-A51	Cell culture^a^	IgG1
CD14	Monocytes	MM61A	VMRD^b^	IgG1
CD25	Activated (IL-2Rα)	IL-A111	Cell culture^a^	IgG1
MHCII	Macrophages, dendritic cells, B cells, activated T cells	IL-A21	Cell culture^a^	IgG2a
WC3	B cells	CC37	Cell culture^a^	IgG1
WC1	γδ T cells	IL-A29	Cell culture^a^	IgG1
Goat anti-mouse		IgG-FITC	Calbiochem	IgG

a Monoclonal antibodies obtained from cell culture were derived from hybridomas sourced from the International Livestock Research Institute in Kenya.

b VMRD, Veterinary Medical Research and Development Inc.

### Genotyping Assays

**Figure 1:**
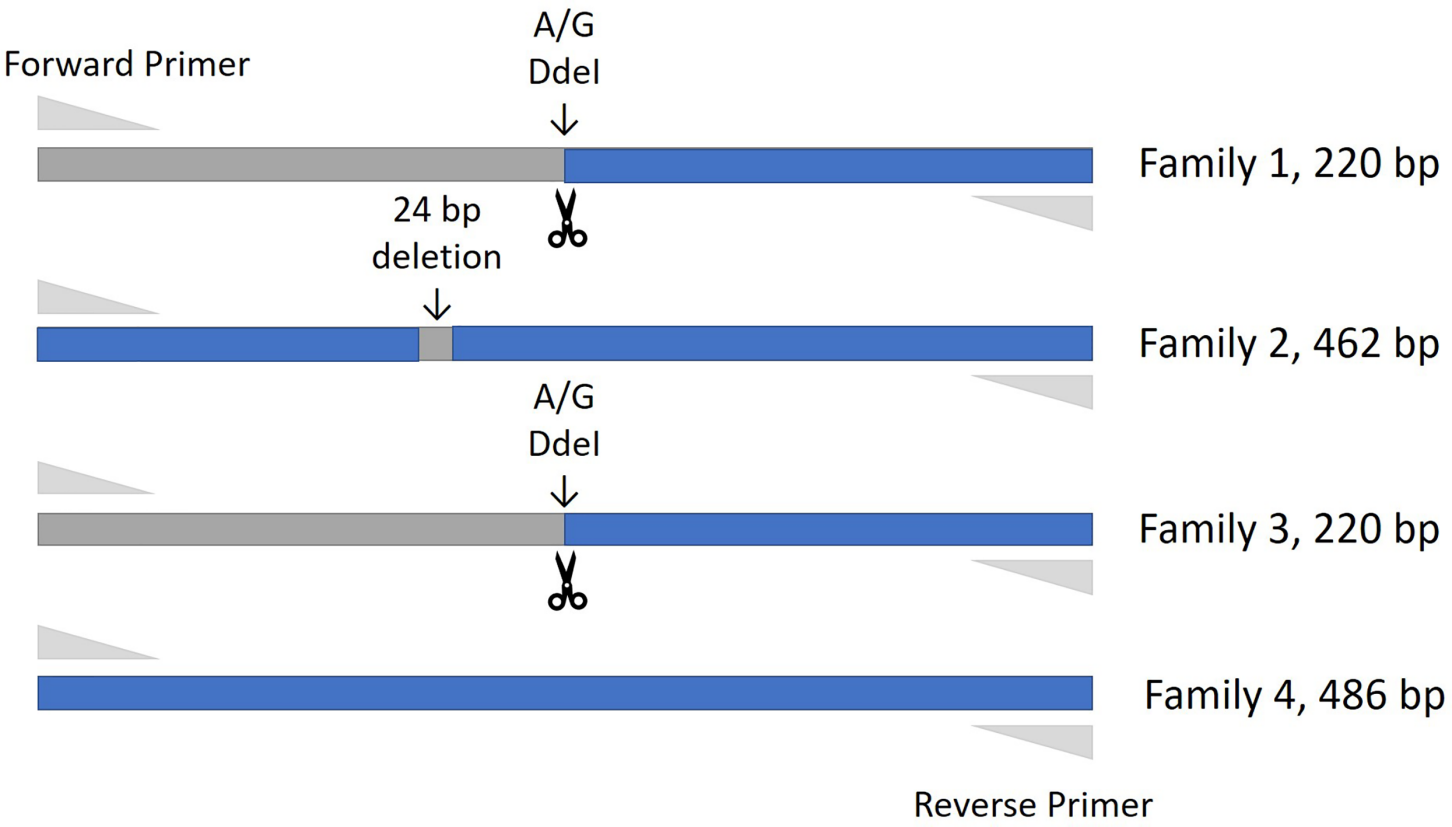
Amplification fragment sizes obtained by the genotyping assay for each of the PTPRC families. The restriction site enzyme DdeI was used to cut the *Bos taurus* (Family 1 & Family 3) reference sequence at location 68,989. Neither the Family 2 nor Family 4 alleles are cut at this location, and these alleles are differentiated by the 24 bp deletion that is the main characteristic of the Family 2 allele.

Thirty-four cattle were genotyped for *PTPRC* families using a restriction-enzyme fragment length-polymorphism (RFLP) assay that differentiated Family 1 and 3 together (220 bp amplicon - taurine and African taurindicine families), from Family 2 (462 bp - indicine), from Family 4 (486 bp - indicine). Accession numbers of publicly available sequences are shown in **Table 2**. Genotyping and sequencing assays assessed the region of *PTPRC* previously identified as exon-9 by Ballingal et al. (2), but which we now consider to most likely correspond with exon-5 or exon-6 (data not shown). The distinguishing features of the 4 families are shown in **Table 3**. We used a modification of their genotyping assay using the primers *CD45ex9_F*: TCCTGGGGCTATTTTTGTTGGTGTT and *CD45ex9_R*: AGGCTGCTCCGAGGTCACCA, with annealing temperature of 59°C, and an expected fragment size of 486 bp. The restriction site enzyme DdeI was used to cut only the *B. taurus* (Family 1 & Family 3) reference sequence at location 68,989. Genotyping was conducted by capillary electrophoresis using a 3130 XL Genetic Analyzer (Thermofisher, Australia). The amplicons generated for Family 1 and Family 3 were the shortest, at 220 bp, whereas Family 2, with the 24-bp deletion, is 462 bp, and Family 4 is the complete amplicon from forward to reverse primer of 486 bp (**Figure 1**).

**Table 2:** Accession numbers and references for nucleotide sequences used in this study.

Accession No.	Species	Exon/Region	Genome Scaffold	Reference
NC_037343.1 (77540526-77670102)	*Bos taurus*		ARS-UCD1.2 Chromosome 16	NCBI Nucleotide
NC_032665.1 (75903959-76032820)	Bos *indicus*		Bos_indicus_1.0 Chromosome 16	
NC_040091.1 (76794293-76923526)	Bos *taurus* x *indicus*		UOA_Brahman_1 Chromosome 16	
AJ278876	*Bos indicus*	Partial Exon 9		Ballingal et al. (2)
AJ278877	*Bos indicus*	Partial Exon 9		
AJ278878	*Bos indicus*	Partial Exon 9		
AJ278879	*Bos indicus*	Partial Exon 9		
AJ400864	*Bos taurus*	Partial mRNA PTPRC gene		

**Table 3:** Major discriminating features of nucleotide sequence used for defining the four distinct *PTPRC* families.

Family	Constant Variant Nucleotides	Insertions or deletions	Genotype in RFLP assay (fragment length)
Family 1 Taurine	Reference sequence	Reference sequence	220 bp
Family 2 Indicine	G<A 68,992	24 bp deletion 68,932	462 bp
	T<A 68,995		
	A<G 69,001		
	(shared Family 2 & 4)		
	plus		
	2 unique SNP		
	G<A 68,876		
	T<A 68,964		
Family 3 Taurindicine	9 unique SNP AG<TT 68,798-9 G<A 68,850	ACA insertion at 68,895	220 bp
		An insertion at 68,761	
		4 bp deletion at 68,792	
	G<A 68,852		
	A<G 68,865		
	G<A 68,894		
	T<A 68,897		
	C<G 68,899		
Family 4 Indicine	G<A 68,992	Nil	486 bp
	T<A 68,995		
	A<G 69,001		
	(shared Family 2 & 4)		
	Plus		
	3 unique SNP		
	G<C 68,890		
	A<G 68,928		
	A<G 68,930		

### Statistical Analysis

All statistical analysis was conducted using R version 4.0.3 [(16) R Core Team, 2018]. Data consisted of 14 or 15 successive time-series observations for each variable for each individual. Only a subset of samples from resistant and susceptible animals had originally been subjected to IgG quantification, so the representation of each of the genotypes was uneven, with some genotypes missing completely. Therefore, only those animals with 220/220 (*n* = 3) and 462/462 (*n*=7) genotypes were included in the analysis for IgG1 and IgG2. All dependent variables were checked for normality by plotting as histograms and application of the Shapiro-Wilk test of normality. Variables with non-normal distributions were tested for compliance after natural log and square root transformations, and if these did not yield normally distributed data, they were then transformed using the Johnson family of distributions using the “ls” procedure from the R package “jtrans” (version 0.2.1). Given the highly skewed time-responses of IgG1 and IgG2, only the distributions of the residuals of the GAMs were checked and all were found to approximate normal distributions. Time was expected to be an important explanatory variable, but there was no *a priori* reason to expect any particular response function for any of the dependent variables over time. Therefore, a generalized additive model was used, with time as a smoothed effect, the allele dosage as a fixed effect (for each of the three alleles, any animal can have the value 0,1,2), and individual animal as a random effect. The R function “gam” from the package “mgcv” (version 1.8-33) was used (17), and models were tested using the “gam.check” function (18). Residuals were plotted for each model and checked for deviations from normality. Estimates of *p*-values are presented in tables as obtained from the models, but a statistical significance level (α) was set at 0.00083, consistent with Bonferroni correction for testing of 60 variables. For the re-analysis of resistance to ticks, a similar approach was taken to make more efficient use of the non-summarized time-series data.

## Results

The most frequent allele was the 462, indicine Family 2, with a relative frequency of 0.47 (32/68 possible alleles), followed by the taurine Family 1/3 allele 220 at 0.34 (23/68 possible alleles), with the 486 allele of the indicine Family 4 being least frequent at 0.19 (13/68 possible alleles). The distribution of genotypes and alleles was uneven, the most common genotype being 462/462, the indicine Family 2 (**Table 4**, 10/34 animal genotypes). However, the observed frequencies of genotypes did not differ from expectations under Hardy-Weinberg equilibrium (**Table 4**, χ^2^ = 3.314, *p* > 0.1).

**Table 4:** *PTPRC* allele and genotype frequencies.

Allele	Allele Count	Allele Frequency	Genotype	Genotype Count		Expected Genotype Frequency	Expected Genotype Count	χ^2^	*p*-value
220	23	0.34	D220/D220	5		0.11	4	3.314, df = 3	> 0.1
462	32	0.47	D220/D462	7		0.32	11		
486	13	0.19	D220/D486	6		0.13	4		
			D462/D462	10		0.22	8		
			D462/D486	5		0.18	6		
			D486/D486	1		0.037	1		
**Genotype**		**Controls**	**Medium Resistance**	**Resistant**	**Susceptible**		**Total**		
220/220		2	0	1	2		5		
220/462		1	6	0	0		7		
220/486		0	4	1	1		6		
462/462		1	5	2	2		10		
462/486		1	3	1	0		5		
486/486		0	1	0	0		1		
Total		5	19	5	5		34		

Part A: overall allele and genotype frequencies and assessment of Hardy-Weinberg equilibrium of alleles and genotypes. Part B: genotypes according to their resistance or experimental status. (Control animals were not infested; the 5 animals with the lowest and highest tick counts were designated Resistant and Susceptible respectively, and the remainder (n=13) were designated as medium).

**Figure 2:**
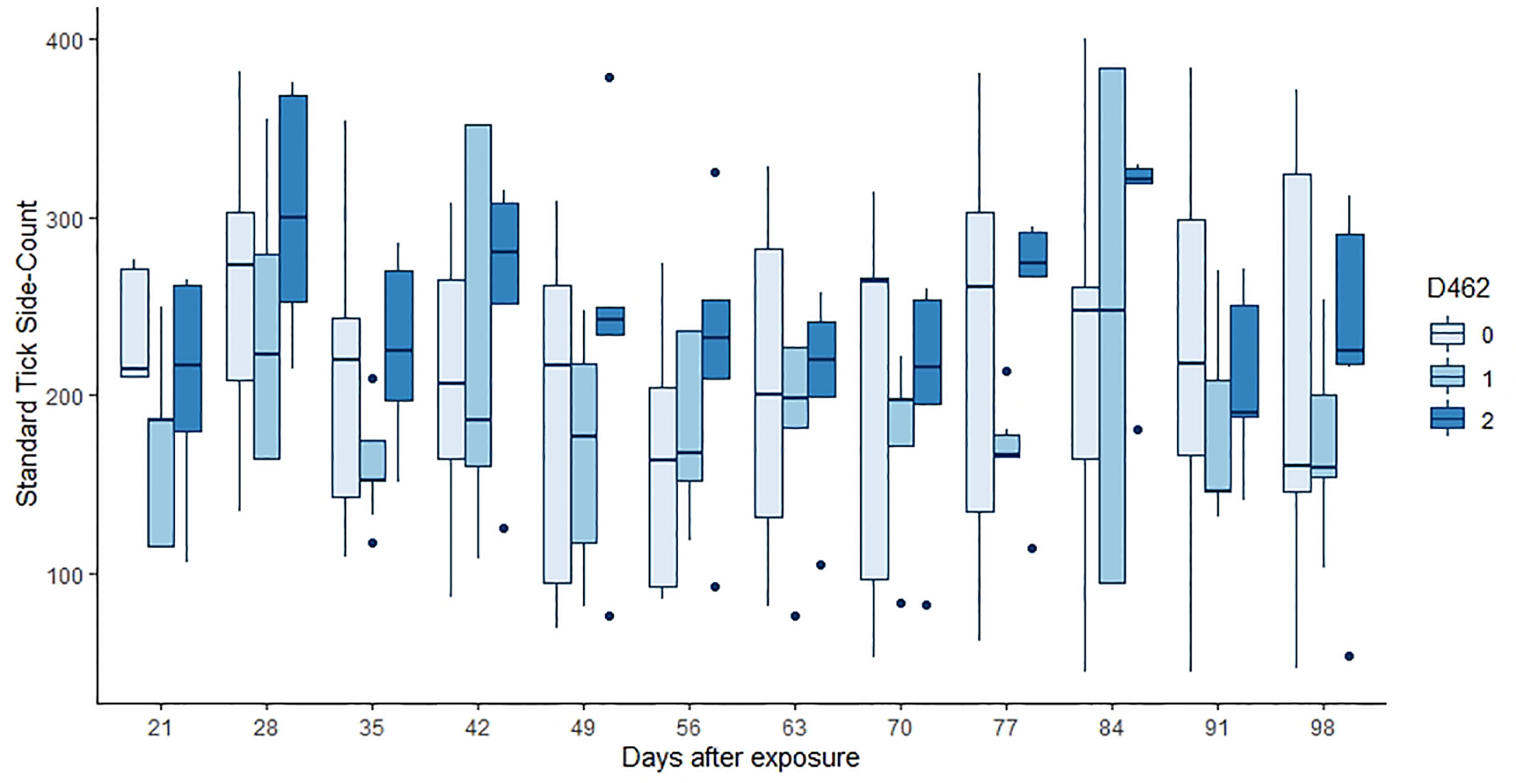
Tick counts by days after exposure, commencing at 21 d post infestation and continuing for 11 weeks. Data for the number of copies of the 462 allele are shown, those animals without the allele in pale blue, and animals that were 462/462 in the darkest blue. Neither the count day nor the allele dose were significant in the GAM (*p* > 0.00083, **Table 5**).

Neither tick burden nor resistance category was significantly influenced by the *PTPRC* genotype. Linear regressions of total or median tick count against genotype were not significant (*p* = 0.46, 0.64, 0.74 for the 222, 462 and 486 genotypes respectively). The GAMs for tick count considered each of 12 weekly timepoints for each of 30 animals, commencing at three weeks after initial infestation. Neither the effect of (smoothed) time nor of dosage of any of the alleles was significant (*p* > 0.00083, **Figure 2** and **Table 5**).

**Table 5:** Summary of GAM outputs for each of the models for tick count and each of the variables for which the main effect of allele frequency was considered to be statistically significant (*p* < 0.00083).

Outcome variable	Transformation	Explanatory variable	Intercept	Effect estimate	*t*	*p*-value	s(time) *F*-value	S(time) *p*-value	Deviance explained
Tick Count (ticks)	None needed	Allele 220	220.978	-16.038	-2.618	0.00928	1.83	0.0536	7.8%
		Allele 462	199.867	11.344	2.002	0.0461	1.807	0.0572	6.9%
		Allele 486	205.721	5.612	0.621	0.535	1.57	0.114	5.5%
White cell count (cells ×10^3^/mm^3^)	Johnson	Allele 220	0.23839	-0.32141	-5.777	1.39e-08	11.1	<2e-16	20.6%
		Allele 462	-0.04420	0.08412	1.570	0.117	10.52	<2e-16	15.3%
		Allele 486	-0.11413	0.24091	4.112	4.63e-05	10.78	<2e-16	17.9%
Red cell count (cells ×10^6^/mm^3^)	sqrt	Allele 220	2.80477	-0.04098	-3.092	0.00211	12.02	3.53e-07	9.3%
		Allele 462	2.71928	0.07549	6.295	7.08e-10	12.74	<2e-16	14.7%
		Allele 486	2.80425	-0.04905	-3.587	0.000369	12.21	<2e-16	9.9%
PCV (%)	Johnson	Allele 220	0.13281	0.13281	2.215	0.0272	7.408	1.08e-06	8.9%
		Allele 462	-0.20056	0.31662	5.753	1.59e-08	7.696	8.7e-07	14.1%
		Allele 486	0.17799	-0.24550	-3.948	9.08e-05	7.548	1.24e-06	10.9%
Hb (g/dl)	log*_e_*	Allele 220	2.461282	-0.018942	-2.408	0.0164	1.128	0.361	3.1%
		Allele 462	2.418265	0.039476	5.52	5.65e-08	1.178	0.319	7.9%
		Allele 486	2.464186	-0.028318	-3.502	0.000507	1.148	0.343	4.4%
CD8 (% gated cells)	Johnson	Allele 220	3.66767	-0.12974	-3.753	0.000197	29.96	<2e-16	34.6%
		Allele 462	3.50044	0.10380	3.21	0.00142	29.76	<2e-16	34.1%
		Allele 486	3.57392	0.01088	0.3	0.764	29.16	<2e-16	32.6%
IgG1 – gut membrane – OD	None	Allele 220	1.25063	-0.16825	-3.711	0.000281	27.3	<2e-16	40.6%
IgG1 – gut soluble – OD	None	Allele 220	0.80045	-0.04853	-4.221	3.96e-05	79.52	<2e-16	40.6%
IgG1 – salivary soluble – OD	None	Allele 220	0.73197	-0.11101	-4.081	6.61e-05	30.54	<2e-16	48.7%

In all cases, the model includes measurement time as a smoothed variable, allele dosage as a fixed effect with three levels (that represent the number of copies of that allele that the individual has: 0,1,2) and animal ID as a random effect. Data have not been back-transformed – model intercepts and effect estimates represent the intercept and effect sizes on the transformed data. Results for the full set of outcome variables are shown in **Table S1**.

**Figure 3:**
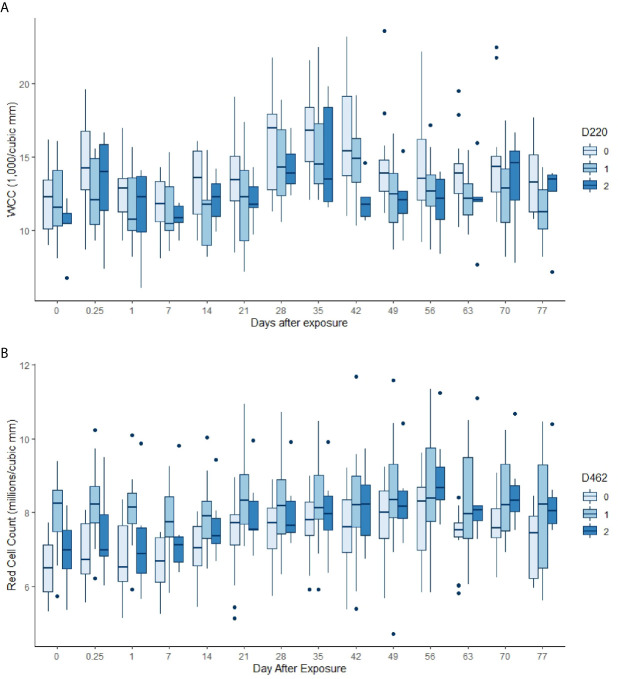
**(A)** White cell counts by day, commencing pre-infestation and continuing for 11 weeks. Data for the 220 allele are shown, those animals without the allele in palest blue, and animals that were 220/220 in the darkest blue. Both day and the allele dose were highly significant in the GAM (*p* < 0.00083, **Table 5**). **(B)** Red cell counts by day, commencing pre-infestation and continuing for 11 weeks. Data for the 462 allele are shown, those animals without the allele in palest blue, and animals that were 462/462 in the darkest blue. Both day and the allele dose were highly significant terms in the GAM (*p* < 0.00083, **Table 5**).

**Figure 4:**
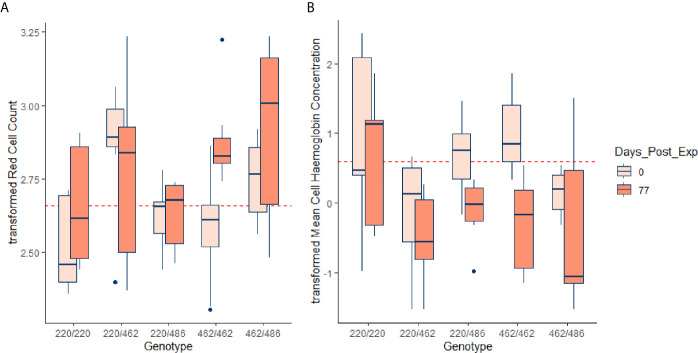
Transformed RCC (tRCC - **A**) and transformed MCH (tMCH - **B**) for the initial (pre-infestation) and end (77 d post-infestation) time points, for each of the genotypes. Horizontal dotted lines in red are the mean pre-infestation values for each of the transformed variables. For RCC, the effect of 462 allele dosage was highly significant in the GAM (*p* < 0.00083, **Table 5**) but for MCH the effect approached significance (*p* = 0.00726, **Table S1**).

**Figure 5:**
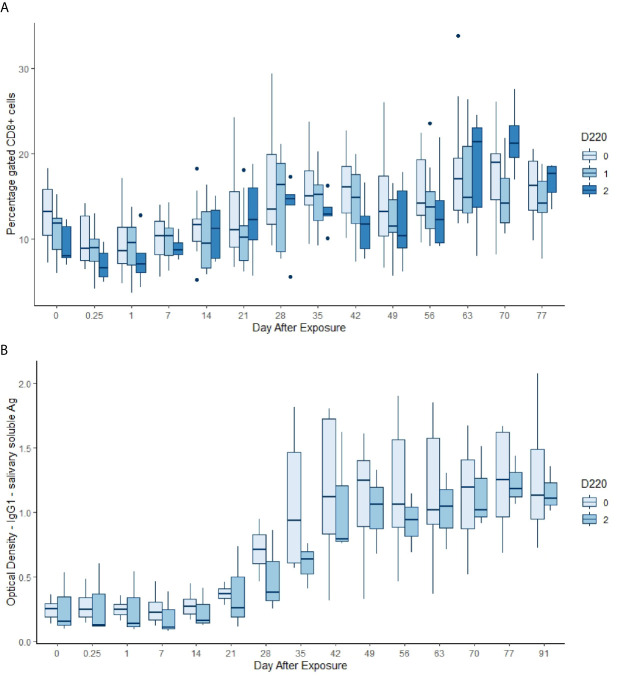
**(A)** Gated percentage of CD8+ cells by day, commencing pre-infestation and continuing for 11 weeks. Data for the 220 allele are shown, those animals without the allele in palest blue, and animals that were 220/220 in the darkest blue. Both day and the allele dose were highly significant in the GAM (*p* < 0.00083, **Table 5**). **(B)** IgG1 optical density (OD) in response to soluble salivary tick Ag by day, commencing pre-infestation and continuing for 15 weeks. Data for the 220 allele are shown and those animals without the allele (pale blue) are all 462/462 (dark blue). Both day and the genotype were highly significant terms in the GAM (*p* < 0.00083, **Table 5**).

Almost all the immunological and haematological assay results were significantly affected by time (**Table 5**, **Table S1** and **Figures S1**–**S3**). Only Hb, platelet count and the response to larval soluble Ag did not vary significantly (*p* > 0.00083) over time. White cell count (WCC) was significantly affected by the doses of alleles 220 and 486. Each dose of allele 220 decreased WCC (*p* = 7.08 × 10^-10^), whereas each dose of allele 486 increased WCC (*p* = 4.63 × 10^-5^, **Figure 3A**). Red cell count (RCC) increased significantly (*p* = 1.39 × 10^-8^, **Figure 3B**) with each dose of the 462 allele and decreased significantly with each dose of the 486 allele (*p* = 0.000369). PCV and Hb followed this same pattern of significant increase with each dose of the 462 allele and significant reduction with each dose of the 486 allele (**Table 5**). For the red blood cell variables, there were distinct response patterns for 462 heterozygotes and 462/462 homozygotes (**Figure 4**). Among the immunolabelled cells, only CD8^+^ cells were significantly associated with allele, being reduced in cattle with each additional copy of the 220 allele (*p* = 0.000197, **Figure 5**). Immunoglobulin responses were affected by genotype; 220/220 animals had consistently lower IgG1 in response to tick Ag than the 462/462 animals. Most of the models failed to explain a large proportion of the deviance – with the best model explaining 48% and the worst model explaining 4% of the deviance.

## Discussion

The study on which this project is based (10, 11) was intended to contrast local and systemic immune responses and haematology between cattle of high resistance and those of low resistance to tick infestations. An incidental finding of the original studies was that highly resistant animals were less likely to have detectable CD45^+^ or CD45RO^+^ cells in skin (10). However, that observation was based on an extreme-group comparison of the 6 most resistant and 6 least resistant animals. In the present study, we genotyped *PTPRC* (CD45) for all the original animals in the trial and found that although there was no significant relationship between tick count and the dosage of any one of the three differentiable alleles, large differences in erythrocyte, leukocyte and humoral responses were observed among *PTPRC* genotypes: the indicine Family 2 (462) allele was associated with a more robust erythron; the “taurindicine” Family 1 allele (220) was associated with lower leukocyte count, lower % gated CD8^+^ cells, and lower IgG1 recognition of tick-specific Ag. Given that these alleles are believed to have tick-resistant and tick-susceptible origins respectively, there is some potential confounding of the apparent allelic effects by alleles at other loci that are in linkage disequilibrium (LD) with them. The Santa Gertrudis breed was selected for this study intentionally to reduce confounding by genetic background. The breed was established in Texas about 100 years ago as a hybrid between *B. taurus* and *B. indicus* cattle, so it is expected that over 30-40 generations of breeding LD should have been reduced among the linked genes and eliminated among the unlinked genes. It follows that caution is required in extrapolating from contrasts among the genotypes in this study to contrasts between indicine and taurine animals from previous studies. It cannot be inferred that differences between *B. indicus* and *B. taurus* cattle can be attributed to variation in *PTPRC* genotype, nor that *PTPRC* genotype is necessarily consistent in populations of *B. taurus* and *B. indicus* cattle. Our unpublished sequence and genotyping data suggest that Brahman cattle in Australia are diverse and include members of all four families, whereas Holstein-Friesian cattle seem to be almost exclusively taurine Family 1.

The most pronounced differences among genotypes were in the variables relating to red blood cells. Cattle with the indicine Family 2 allele for *PTPRC* (462) had higher RCC, PCV and Hb. The Family 2 heterozygotes had significantly higher RCC than the Family 2 homozygotes during the pre-infestation and early infestation periods, but by 11 weeks the homozygote was also high. Similar patterns were noted for PCV and Hb. At the end of the study period, MCH was lowest in Family 2 homozygotes, which, taken with the increase in RCC in these animals, is consistent with a stronger regenerative response to blood loss. Red cell counts have previously been reported to be higher in tick-infested indicine than taurine cattle, in the absence of *Babesia* and *Anaplasma* haemoparasites (15), and greater resistance to reduction in erythrocyte counts of *B. indicus* cattle that have been exposed to *Babesia* has also been demonstrated (19). All nucleated haematopoietic cells express CD45, the dominant isoforms being RO and RB (3). Although most investigations on CD45 function have focused on immune signalling, it has been shown that CD45 is an important regulator of splenic erythropoiesis (20). Although the bulk of erythropoiesis occurs in the bone marrow, splenic erythropoiesis, supported by red pulp macrophages (RPM) makes an important contribution to the expansion of the erythron in response to diverse stressors including hypoxia, endotoxins, bacterial and viral infections. Mice that are deficient in CD45 show abnormal erythropoiesis and accumulate progenitor forms of erythrocytes (20). It has also been shown that CD45 is a negative regulator of erythropoietin-dependent haematopoiesis through its inhibition of Janus kinase (JAK) signalling pathways (21). Therefore, there are several mechanisms by which variation in CD45 genotype could influence haematopoiesis, and the observations from our study are consistent with the pathogen-driven selection hypothesis advanced by Ballingal et al. (2).

Cattle with the taurine Family 1 (220) allele for *PTPRC* had lower WCC and lower gated percentages of CD8^+^ cells (T cytotoxic cells) in circulation. Immunoglobulins specific to three of five tick Ag mixtures differed highly significantly between homozygotes of the Family 1/3 (220) and the Family 2 (462) genotypes. Among the cell proliferation assays conducted in our study, genotype did not have a significant effect, using α corrected for multiple comparisons to 0.00083. However, several of the GAMs estimated *p*-values approaching this level (Family 1/3 allele 220: *p* = 0.00174 for ConA stimulation, and *p* = 0.00181 for larval soluble Ag). Diverse leukocytic responses to tick infestation have been reported in tick-infested cattle of indicine and taurine origins. Rechav (22) reported that Simmental (*B. taurus*) cattle had higher leukocyte counts than Brahmans (*B. indicus*) when infested with diverse species of African ticks. We previously found a similar result in a contrast between tick-infested Holstein-Friesian (*B. taurus*) and Brahman (*B. indicus*) cattle (15). Immunoglobulin production in response to tick Ag has been shown to differ between taurine and indicine cattle exposed to ticks although the directions of the associations have not been consistent among studies and experimental conditions (15, 23). Rocha Garcia et al. (24) confirmed that there were clear differences between taurine and indicine cattle in their ability to recognize and respond to tick Ag. The lymphoproliferative, phagocytosis and oxidative burst activity of neutrophils and monocytes differs between indicine and taurine cattle, each responding differently to co-culturing with *R. microplus* salivary gland extract (25). Ramachandra and Wikel (26) found substantial differences in taurine and indicine leukocyte biology – T cells from *B. indicus* cattle had a stronger proliferative response to ConA and peripheral blood mononuclear cells from *B. indicus* cattle produced more IL-1 in response to lipopolysaccharide (LPS). Given the many mechanisms by which CD45 is known to modulate leukocyte proliferation and cytokine responses to various stimuli (4, 21), the divergent leukocyte biology evident in animals of the different genotypes in our study is not surprising.

The immunological observations used in our study were selected with a view to better understanding the mechanisms underlying the differences in host resistance to tick infestation rather than for the characterization of the complete immunological phenotypes of animals of each of the *PTPRC* genotypes. As such, we have an incomplete set of observations on a relatively small dataset of animals that is not balanced by genotype. However, our population does have the advantage of being drawn from a breed in which we expect some of the confounding effects of linkage disequilibrium to have been reduced or eliminated. The effects of CD45 are mediated largely by variation in isoform expression and glycosylation rather than by variable ligand binding or variable enzyme expression, and most of the clinically relevant polymorphisms in humans influence isoform expression (4). At present there is not enough information on the full genomic sequence variants of *PTPRC* in cattle or isoform expression variants to confidently relate the cattle genotypic families to any studies on human or murine variants of *PTPRC*. Nonetheless, it seems safe to conclude that variation in *PTPRC* is likely to contribute to variation in the profiles and functions of leukocytes and erythrocytes of cattle. In human medicine, CD45 isoform expression is used as an important component of clinical immunological profiles (27). In cattle, there are relatively few reports on its application, although it has been used as one of several markers of immune response to mastitis (28) and rumen fluke (29), among others. In our study, *PTPRC* polymorphism was strongly associated with divergent erythrocytic, leukocytic and humoral responses to tick infestation. The extent to which this might be useful to aid in the selection of adapted cattle will depend on better knowledge of the variants in populations of cattle, the link between polymorphism of *PTPRC*, form and function of CD45, and possible interactions with other genes.

## Data Availability Statement

The raw data supporting the conclusions of this article will be made available by the authors, without undue reservation.

## Ethics Statement

The animal study was reviewed and approved by University of Queensland Animal Ethics - Production and Companion Animals Committee.

## Author Contributions

NJ – conceived study, coordinated original field work, undertook original field work, undertook data analysis, and drafted paper. DC – undertook genetic analysis. EP – undertook original field work, and conducted laboratory work including immunological assays. EM – undertook genetic analyses. CC – carried out immunological assays. LJ – conceived study, undertook original field work, and undertook immunological assays. MS – contributed to genetic analyses. AT – conceived study, and coordinated original project. All authors contributed to the article and approved the submitted version.

## Funding

This project was initially funded by the Cooperative Research Centre for Beef Genetic Technologies (Beef CRC). DC was funded by the BBSRC Research Experience Placement Programme (Characterisation of protein tyrosine phosphatase receptor C gene in cattle of divergent phylogeny).

## Conflict of Interest

The authors declare that the research was conducted in the absence of any commercial or financial relationships that could be construed as a potential conflict of interest.
