# Supplementary material for: Allelic Variation in Protein Tyrosine Phosphatase Receptor Type-C in Cattle Influences Erythrocyte, Leukocyte and Humoral Responses to Infestation With the Cattle Tick Rhipicephalus australis
Source: Front Immunol. 2021 Jul 9;12:675979. doi: 10.3389/fimmu.2021.675979 (PMC8300432; doi:10.3389/fimmu.2021.675979)
Supplement: Supplementary file 1 [file DataSheet_1.docx]

| Supplementary Table S1. Summary of GAM outputs for each of the explanatory variables. In all cases, the model includes measurement time as a smoothed variable, allele dosage as a fixed effect with three levels (that represent the number of copies of that allele that the individual has: 0,1,2) and animal ID as a random effect. Significant fixed effects (p < 0.00083) are in bold text. Data have not been back-transformed – model intercepts and effect estimates represent the intercept and effect sizes on the transformed data. | | | | | | | | | |
| --- | --- | --- | --- | --- | --- | --- | --- | --- | --- |
| Outcome variable | Transformation | Explanatory variable | Intercept | Effect estimate | *t* | *p*-value | s(time) *F*-value | S(time) *p*-value | Deviance explained |
| Tick Count (ticks) | None needed | Allele 220 | 220.978 | -16.038 | -2.618 | 0.00928 | 1.83 | 0.0536 | 7.8% |
|  |  | Allele 462 | 199.867 | 11.344 | 2.002 | 0.0461 | 1.807 | 0.0572 | 6.9% |
|  |  | Allele 486 | 205.721 | 5.612 | 0.621 | 0.535 | 1.57 | 0.114 | 5.5% |
| Haematology | | | | | | | | | |
| White cell count (cells ×10^3^/mm^3^) | Johnson | Allele 220 | 0.23839 | -0.32141 | -5.777 | **1.39e-08** | 11.1 | **<2e-16** | 20.6% |
|  |  | Allele 462 | -0.04420 | 0.08412 | 1.570 | 0.117 | 10.52 | **<2e-16** | 15.3% |
|  |  | Allele 486 | -0.11413 | 0.24091 | 4.112 | **4.63e-05** | 10.78 | **<2e-16** | 17.9% |
| Red cell count (cells ×10^6^/mm^3^) | sqrt | Allele 220 | 2.80477 | -0.04098 | -3.092 | 0.00211 | 12.02 | **3.53e-07** | 9.3% |
|  |  | Allele 462 | 2.71928 | 0.07549 | 6.295 | **7.08e-10** | 12.74 | **<2e-16** | 14.7% |
|  |  | Allele 486 | 2.80425 | -0.04905 | -3.587 | **0.000369** | 12.21 | **<2e-16** | 9.9% |
| PCV (%) | Johnson | Allele 220 | 0.13281 | 0.13281 | 2.215 | 0.0272 | 7.408 | **1.08e-06** | 8.9% |
|  |  | Allele 462 | -0.20056 | 0.31662 | 5.753 | **1.59e-08** | 7.696 | **8.7e-07** | 14.1% |
|  |  | Allele 486 | 0.17799 | -0.24550 | -3.948 | **9.08e-05** | 7.548 | **1.24e-06** | 10.9% |
| MCH (g/dl) | Johnson | Allele 220 | 0.003305 | 0.087355 | 1.460 | 0.145 | 12.37 | **<2e-16** | 18.7% |
|  |  | Allele 462 | 0.17699 | -0.14972 | -2.697 | 0.00726 | 12.48 | **<2e-16** | 19.6% |
|  |  | Allele 486 | 0.01209 | 0.09077 | 1.465 | 0.144 | 12.38 | **<2e-16** | 18.7% |
| Hb (g/dl) | log*_e_* | Allele 220 | 2.461282 | -0.018942 | -2.408 | 0.0164 | 1.128 | 0.361 | 3.1% |
|  |  | Allele 462 | 2.418265 | 0.039476 | 5.52 | **5.65e-08** | 1.178 | 0.319 | 7.9% |
|  |  | Allele 486 | 2.464186 | -0.028318 | -3.502 | **0.000507** | 1.148 | 0.343 | 4.4% |
| Platelets (platelets ×10^3^/mm^3^) | Johnson | Allele 220 | -0.03433 | 0.03910 | 0.598 | 0.550 | 2.744 | 0.00251 | 6.1% |
|  |  | Allele 462 | -0.03775 | 0.03926 | 0.643 | 0.520 | 2.745 | 0.00249 | 6.1% |
|  |  | Allele 486 | 0.04251 | -0.09028 | -1.334 | 0.183 | 2.747 | 0.00248 | 6.3% |
| Flow cytometry | | | | | | | | | |
| CD3 (% gated cells) | None needed | Allele 220 | 51.1456 | 0.3663 | 0.666 | 0.506 | 9 6.03 | **<2e-16** | 11.1% |
|  |  | Allele 462 | 51.0756 | 0.4175 | 0.816 | 0.415 | 6.189 | **<2e-16** | 11.1% |
|  |  | Allele 486 | 51.8969 | -0.9021 | -1.594 | 0.112 | 6.218 | **<2e-16** | 11.5% |
| CD4 (% gated cells) | sqrt | Allele 220 | 2.99317 | -0.01213 | -0.905 | 0.366 | 27.06 | **<2e-16** | 34.0% |
|  |  | Allele 462 | 2.97082 | 0.01849 | 1.484 | 0.138 | 27.14 | **<2e-16** | 34.2% |
|  |  | Allele 486 | 2.990438 | -0.009794 | -0.708 | 0.479 | 27.05 | **<2e-16** | 34.0% |
| CD8 (% gated cells) | Johnson | Allele 220 | 3.66767 | -0.12974 | -3.753 | **0.000197** | 29.96 | **<2e-16** | 34.6% |
|  |  | Allele 462 | 3.50044 | 0.10380 | 3.21 | 0.00142 | 29.76 | **<2e-16** | 34.1% |
|  |  | Allele 486 | 3.57392 | 0.01088 | 0.3 | 0.764 | 29.16 | **<2e-16** | 32.6% |
| CD14 (% gated cells) | Johnson | Allele 220 | 0.02075 | 0.02048 | 0.367 | 0.714 | 14.63 | **<2e-16** | 22.2% |
|  |  | Allele 462 | 0.15141 | -0.15275 | -2.966 | 0.00317 | 14.9 | **<2e-16** | 23.6% |
|  |  | Allele 486 | -0.05803 | 0.16577 | 2.904 | 0.00386 | 14.89 | **<2e-16** | 23.5% |
| CD25 (% gated cells) | Johnson | Allele 220 | 0.041290 | -0.004522 | -0.080 | 0.936 | 17.25 | **<2e-16** | 25.3% |
|  |  | Allele 462 | -0.01965 | 0.07569 | 1.443 | 0.15 | 17.33 | **<2e-16** | 25.7% |
|  |  | Allele 486 | 0.08748 | -0.08813 | -1.516 | 0.1302 | 17.34 | **<2e-16** | 25.7% |
| CD45RO (% gated cells) | None needed | Allele 220 | 28.9297 | 0.5711 | 0.944 | 0.346 | 11.46 | **<2e-16** | 24.3% |
|  |  | Allele 462 | 29.3905 | 0.1812 | 0.321 | 0.749 | 11.43 | **<2e-16** | 24.1% |
|  |  | Allele 486 | 29.9085 | -1.0418 | -1.492 | 0.137 | 11.5 | **<2e-16** | 24.6% |
| WC1 (% gated cells) | log*_e_* | Allele 220 | 1.77918 | 0.01792 | 0.743 | 0.458 | 6.682 | **<2e-16** | 12.2% |
|  |  | Allele 462 | 1.80443 | -0.01714 | -0.764 | 0.445 | 6.685 | **<2e-16** | 12.2% |
|  |  | Allele 486 | 1.790204 | 0.002009 | 0.081 | 0.936 | 6.678 | **<2e-16** | 12.1% |
| WC3 (% gated cells) | None needed | Allele 220 | 20.5646 | -0.3687 | -1.157 | 0.248 | 25.46 | **<2e-16** | 33.0% |
|  |  | Allele 462 | 19.9153 | 0.5235 | 1.768 | 0.0778 | 25.55 | **<2e-16** | 33.2% |
|  |  | Allele 486 | 20.4558 | -0.2507 | -0.761 | 0.447 | 25.41 | **<2e-16** | 32.9% |
| MHC2 (% gated cells) | None needed | Allele 220 | 34.9888 | 0.9073 | 1.42 | 0.156 | 12.23 | **<2e-16** | 19.4% |
|  |  | Allele 462 | 35.2766 | 0.4263 | 0.715 | 0.475 | 12.19 | **<2e-16** | 19.2% |
|  |  | Allele 486 | 36.4348 | -1.4894 | -2.266 | 0.0239 | 12.31 | **<2e-16** | 20.0% |
| Lymphocyte proliferation assays | | | | | | | | | |
| CONA (OD) | sqrt | Allele 220 | 2.23736 | 0.08549 | 3.15 | 0.00174 | 18.06 | **<2e-16** | 25.8% |
|  |  | Allele 462 | 2.33514 | -0.05224 | -2.054 | 0.0405 | 17.86 | **<2e-16** | 24.9% |
|  |  | Allele 486 | 2.31022 | -0.02688 | -0.95 | 0.342 | 17.74 | **<2e-16** | 24.4% |
| Salivary soluble (OD) | Johnson | Allele 220 | 0.01855 | 0.09808 | 1.715 | 0.0869 | 15.14 | **<2e-16** | 23.2% |
|  |  | Allele 462 | 0.080995 | 0.005107 | 0.096 | 0.924 | 15.05 | **<2e-16** | 22.7% |
|  |  | Allele 486 | 0.14675 | -0.11069 | -1.877 | 0.06109 | 15.16 | **<2e-16** | 23.3% |
| Salivary membrane-bound (OD) | Johnson | Allele 220 | 0.15991 | -0.08566 | -2.115 | 0.0349 | 31.43 | **<2e-16** | 38.2% |
|  |  | Allele 462 | 0.106866 | -0.006406 | -0.169 | 0.8658 | 31.13 | **<2e-16** | 37.6% |
|  |  | Allele 486 | 0.04661 | 0.09906 | 2.374 | 0.018 | 31.5 | **<2e-16** | 38.3% |
| Gut membrane-bound (OD) | Johnson | Allele 220 | 1.80838 | 0.01615 | 0.232 | 0.817 | 12.77 | **<2e-16** | 20.2% |
|  |  | Allele 462 | 1.84080 | -0.02811 | -0.434 | 0.665 | 12.77 | **<2e-16** | 20.2% |
|  |  | Allele 486 | 1.80962 | 0.01733 | 0.241 | 0.81 | 12.77 | **<2e-16** | 20.2% |
| Gut soluble (OD) | Johnson | Allele 220 | -0.05968 | 0.06563 | 1.147 | 0.252 | 7.527 | **<2e-16** | 13.1% |
|  |  | Allele 462 | 0.008529 | -0.031145 | -0.584 | 0.559 | 7.511 | **<2e-16** | 13.0% |
|  |  | Allele 486 | 0.002386 | -0.031626 | -0.535 | 0.593 | 7.51 | **<2e-16** | 12.9% |
| Larval soluble (OD) | Johnson | Allele 220 | -0.13083 | 0.20438 | 3.138 | 0.00181 | 0.604 | 0.437 | 2.2% |
|  |  | Allele 462 | 0.12003 | -0.14725 | -2.418 | 0.0160 | 0.599 | 0.439 | 1.34% |
|  |  | Allele 486 | 0.02797 | -0.03676 | -0.542 | 0.588 | 0.592 | 0.442 | 0.2% |
| Immunoglobulin responses (due to smaller sample size, only homozygote 220/220 and 462/462 included in this analysis) | | | | | | | | | |
| IgG1 – gut membrane (OD) | None | Allele 220 | 1.25063 | -0.16825 | -3.711 | **0.000281** | 27.3 | **<2e-16** | 40.6% |
| IgG1 – gut soluble (OD) | None | Allele 220 | 0.80045 | -0.04853 | -4.221 | **3.96e-05** | 79.52 | **<2e-16** | 40.6% |
| IgG1 – larval soluble (OD) | None | Allele 220 | 0.44251 | -0.05506 | -3.275 | 0.00126 | 11.19 | **<2e-16** | 28.5% |
| IgG1 – salivary soluble (OD) | None | Allele 220 | 0.73197 | -0.11101 | -4.081 | **6.61e-05** | 30.54 | **<2e-16** | 48.7% |
| IgG2 – gut membrane (OD) | None | Allele 220 | 0.51050 | -0.04445 | -1.58 | 0.116 | 3.512 | 0.00581 | 10.2% |


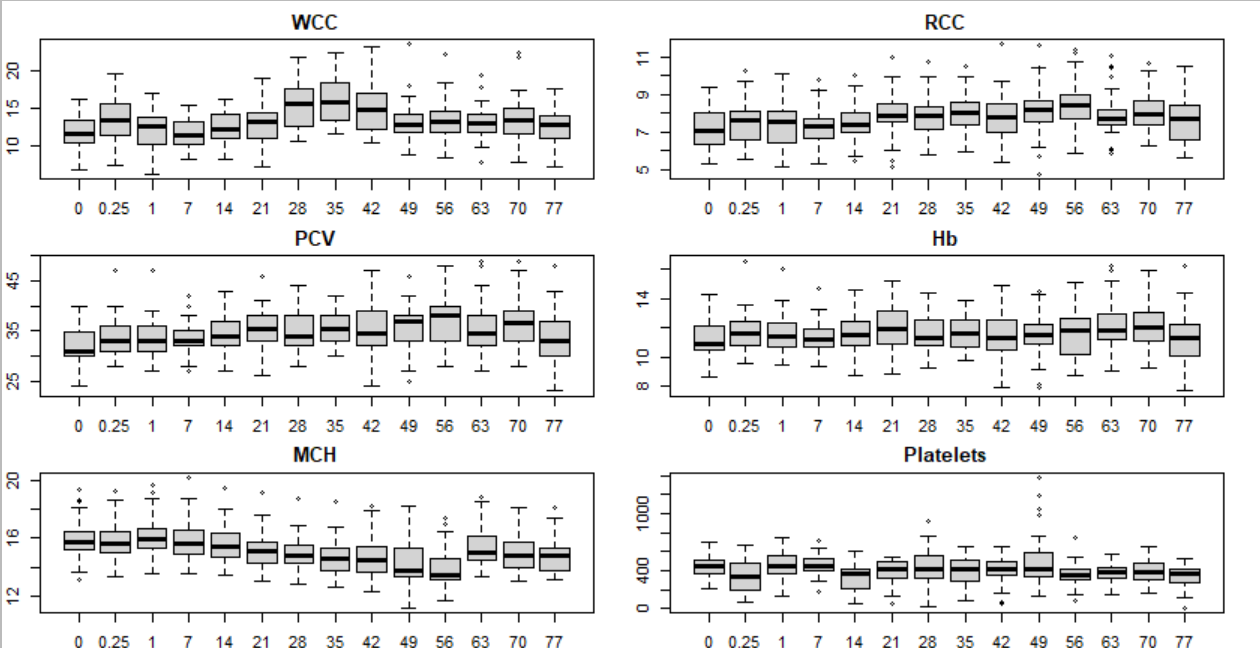


Supplementary Figure S1: Time series box and whisker plots of haematological variables total leukocyte count (WCC – cells × 10^3^/mm^3^), red blood cell count (RCC – cells × 10^6^/mm^3^), packed cell volume (PVC – %), haemoglobin concentration (Hb – g/dl), mean cell haemoglobin (MCH – g/dl), platelets (platelets cells × 10^3^/mm^3^).


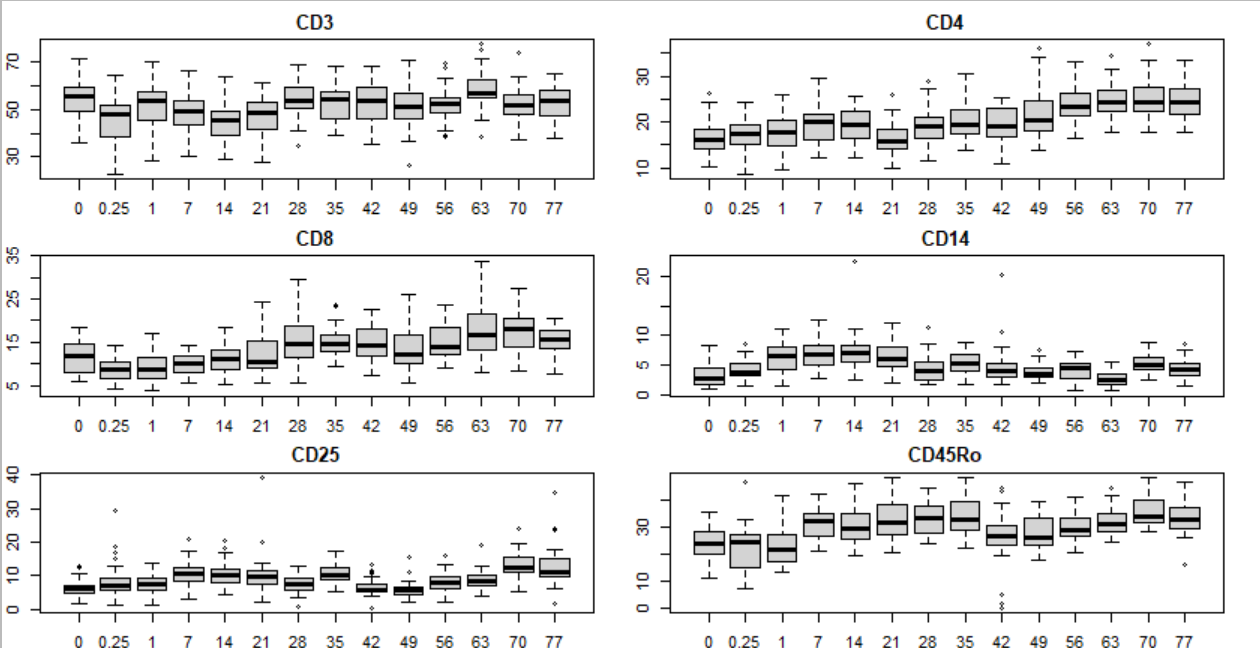

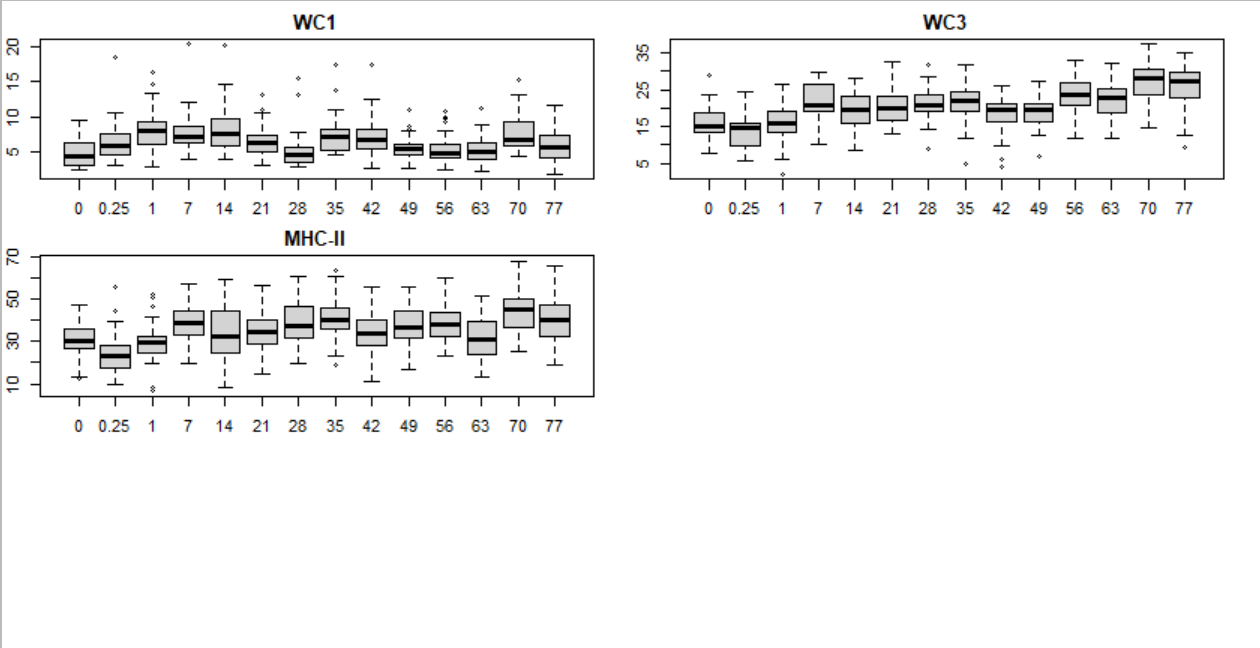


Supplementary Figure S2: Time series box and whisker plots of percentage gated cells belonging to each leukocyte cell subset in peripheral circulation, determined from flow cytometry.


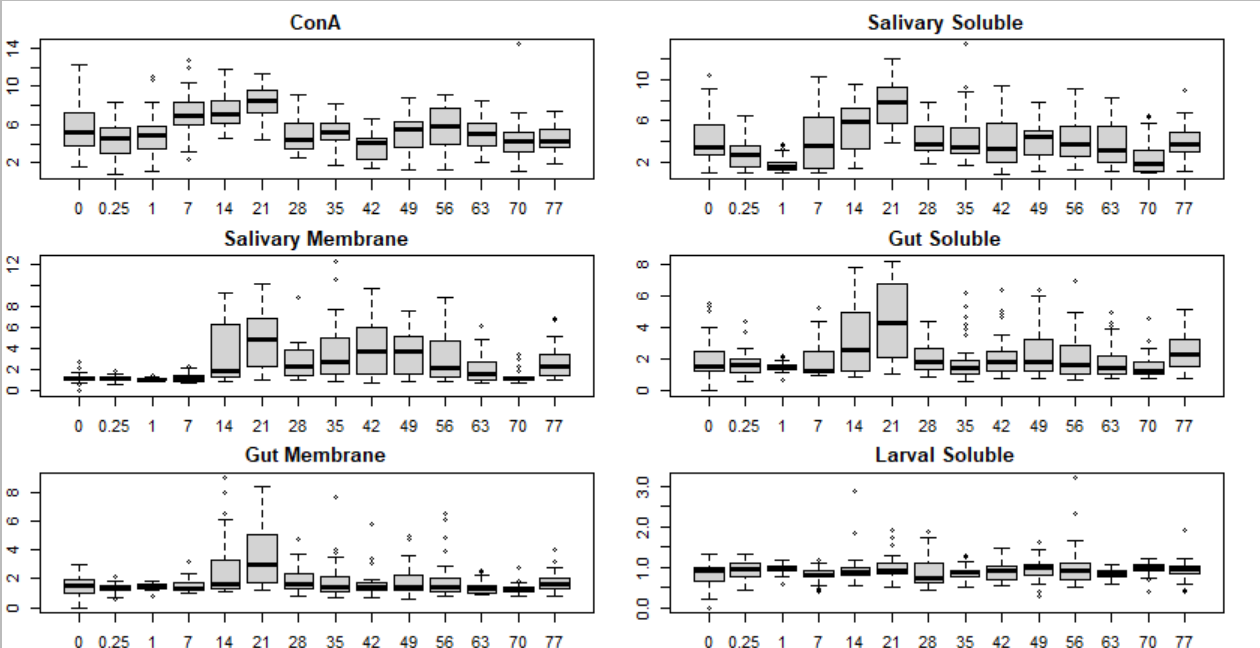


Supplementary Figure S3: Time series box and whisker plots of response to lymphocyte proliferation assays in response to ConA and tick-derived, fractionated Ag.
